# Supplementary material for: Automated model-predictive design of synthetic promoters to control transcriptional profiles in bacteria
Source: Nat Commun. 2022 Sep 2;13:5159. doi: 10.1038/s41467-022-32829-5 (PMC9440211; doi:10.1038/s41467-022-32829-5)
Supplement: Supplementary file 2 — Supplementary Information [file 41467_2022_32829_MOESM2_ESM.pdf]

Supplementary Information

Automated model-predictive design of synthetic promoters to control transcriptional profiles in bacteria

Travis L. LaFleur[1], Ayaan Hossain[2], Howard M. Salis[1,2,3,4] *

[1]Department of Chemical Engineering, [2]Bioinformatics and Genomics, [3]Department of Biological Engineering, [4]Department of Biomedical Engineering, Pennsylvania State University; University Park, Pennsylvania, 16801, United States

*Corresponding author. Email: salis@psu.edu

Table of Contents

Supplementary Figure 1: Replicate Correlations 1

Supplementary Figure 2: Transcription Start Site Profiles. 2

Supplementary Figure 3: Model Predictions on IPTG-inducible Promoters. 3

Supplementary Figure 4: Model Residuals Across Transcription Space. 4

Supplementary Figure 5: Model Accuracy on Selected Non-Repetitive Promoters with 3 Types of Measurements 5

Supplementary Figure 6: Sequence Entropy for 60bp Forward Engineered Promoters 6

Supplementary Figure 7: mRNA Decay Alters RNA Level Measurements 7

Supplementary Figure 8: Hexamer Model Comparison (1-mer vs. 3-mer) 8

Supplementary Figure 9: A Quadratic Non-Linear Free Energy Model 9

Supplementary Figure 10: Oligo Designs and Plasmid Map 10

Supplementary Figure 11: Lagator Extended Model Predictions on Lagator Datasets 11

Supplementary Figure 12: Lagator Extended Model Validation 12

Supplementary Figure 13: Slope-only Model Benchmarking, Residual Distributions 13

Supplementary Figure 14: Flow Cytometry Gating 14

Supplementary Table 1: Model Features 15

Supplementary Table 2: Model Selection 16

Supplementary Table 3: Primers, RNA Adapter, and DNA Fragment Sequences 17

Supplementary Table 4: Model Benchmarking – Slope Only 18

Supplementary Table 5: Model Benchmarking –Intercept Only 19

Supplementary Table 6: Model Benchmarking – Slope and Intercept 20

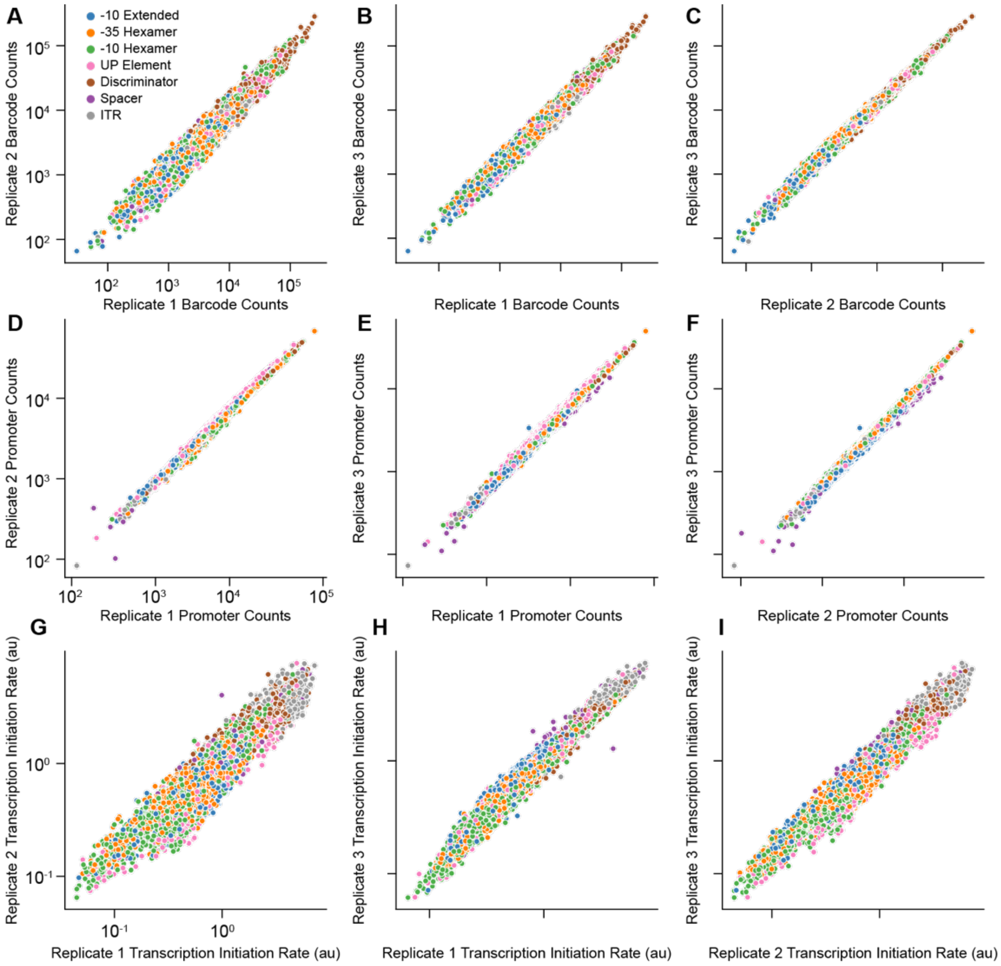

Supplementary Figure 1: Replicate Correlations. RNA read count correlations for (**A**) replicate 1 vs replicate 2, (**B**) replicate 1 vs replicate 3 and (**C**) replicate 2 vs replicate 3 (R^2 = 0.94, 0.97, and 0.99 respectively). DNA read count correlations for (**D**) replicate 1 vs replicate 2, (**E**) replicate 1 vs replicate 3 and (**F**) replicate 2 vs replicate 3 (R^2 = 0.99, 0.99, and 0.99 respectively). Transcription Initiation Rate correlations for (**G**) replicate 1 vs replicate 2, (**H**) replicate 1 vs replicate 3 and (**I**) replicate 2 vs replicate 3 (R^2 = 0.89, 0.94, and 0.97 respectively). Blue dots represent -10 extended promoter variants, orange dots represent -35 hexamer promoter variants, green dots represent -10 hexamer promoter variants, pink dots represent UP element promoter variants, brown dots represent discriminator promoter variants, purple dots represent spacer promoter variants, and grey dots represent ITR promoter variants. Data are provided in **Supplementary Data 1**.

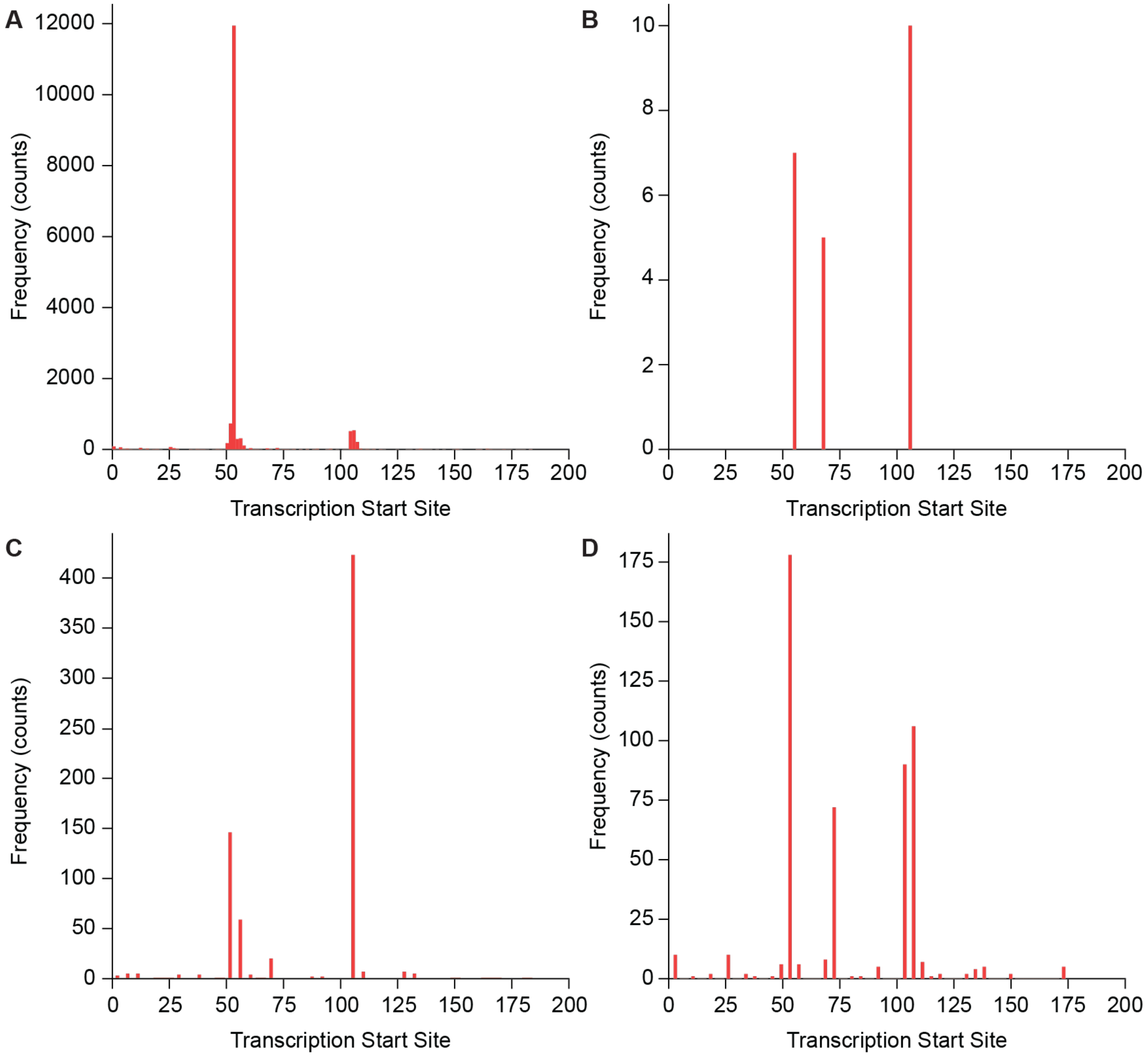

Supplementary Figure 2: Transcription Start Site Profiles. (**A**) The measured TSS distribution of a variant that met all filtering criteria and exhibited a strong on-target peak. (**B**) The measured TSS distribution of a variant that did not meet the minimum read threshold. (**C**) The measured TSS distribution of a variant that had an off-target peak (position ~105) with greater than twice the counts of the on-target peak (position 53). (**D**) The measured TSS distribution of a variant with multiple strong TSS peaks. Data are provided in **Supplementary Data 1**.

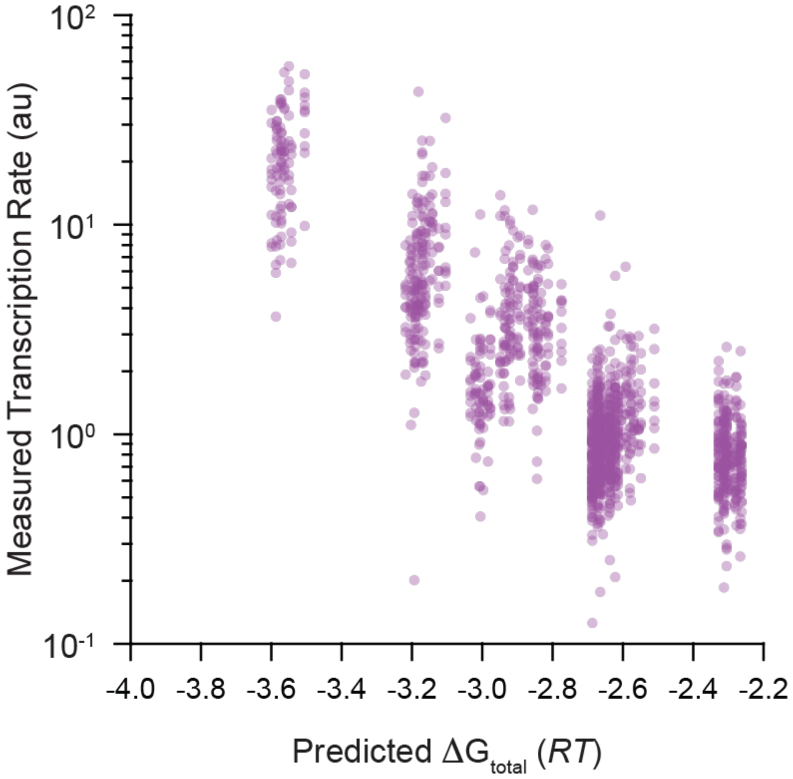

Supplementary Figure 3: Model Predictions on IPTG-inducible Promoters. LaFleur linear model free energy predictions are compare to measured transcription rates for 1493 genome-integrated IPTG-inducible promoters, characterized *in vivo* by Yu et al. with 1 mM IPTG added [17] (R^2 = 0.65, Spearman's ρ = 0.70). Data are provided in **Supplementary Data 1**.

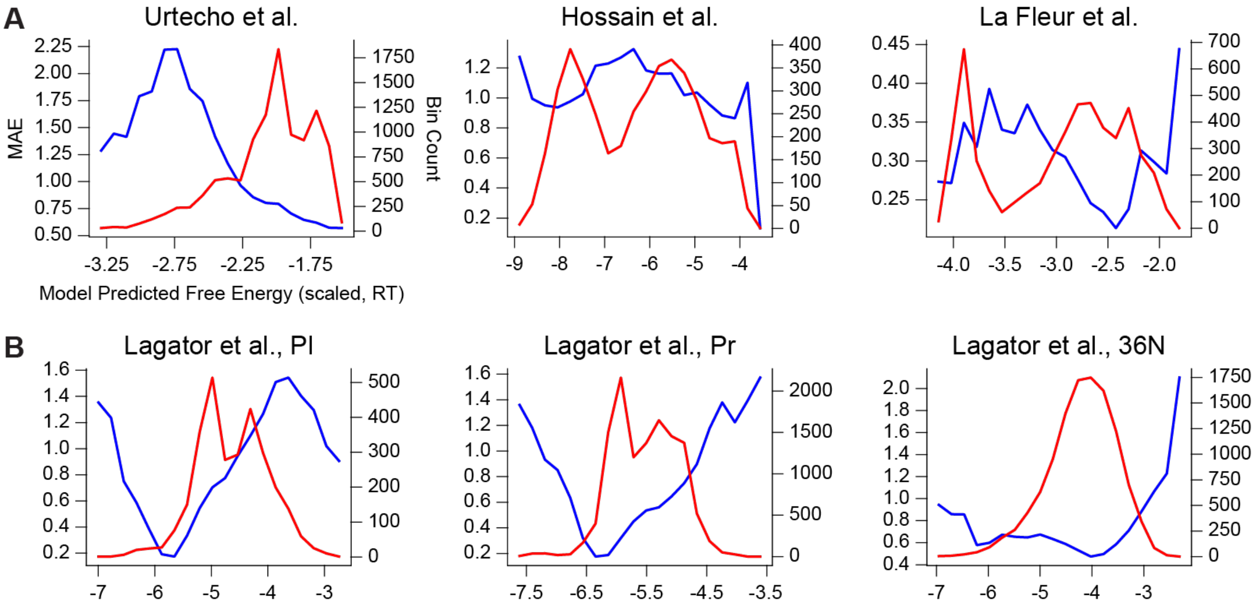

Supplementary Figure 4: Model Residuals Across Transcription Space. (**A**) LaFleur linear model residual distributions across 20 evenly spaced free energy bins for Urtecho et al. (left), Hossain et al. (middle) and LaFleur et al. (right). (**B**) LaFleur linear model residual distributions across 20 evenly spaced free energy bins for the Pl dataset from Lagator et al. (left), Pr dataset from Lagator et al. (middle) and 36N dataset from Lagator et al. (right). Blue lines are MAE values which correspond to the left y-axis, red lines are the number of variants in each bin which correspond to the right y-axis. Data are provided in **Supplementary Data 4**.

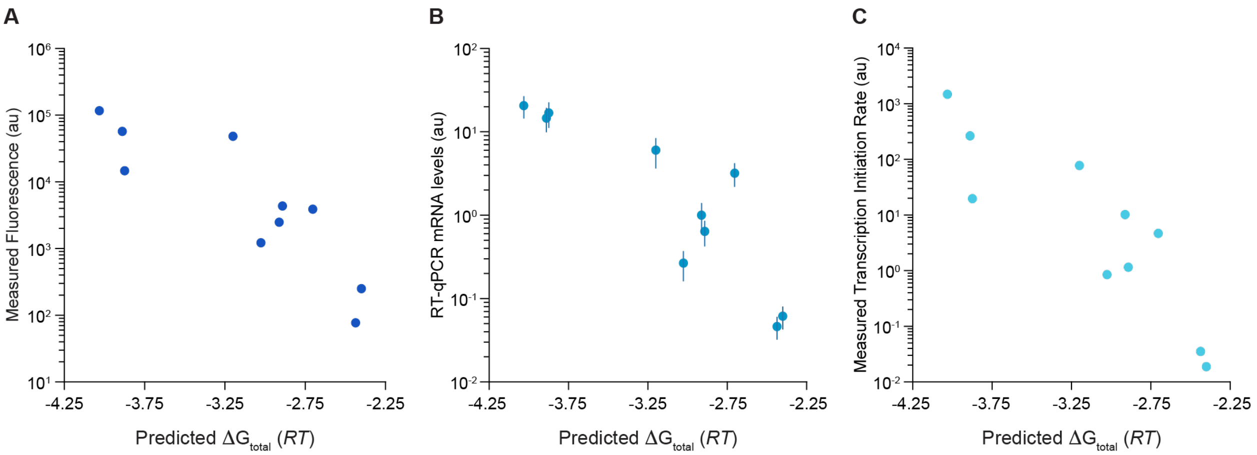

Supplementary Figure 5: Model Accuracy on Selected Non-Repetitive Promoters with 3 Types of Measurements. (**A**) Model predictions are compared to transcription rate measurements for 10 promoters characterized by Hossain et al. using flow cytometry (R^2 = 0.75, Spearman's ρ = 0.87). (**B**) Model predictions are compared to transcription rate measurements for 10 promoters characterized by Hossain et al. using RT-qPCR (R^2 = 0.78, Spearman's ρ = 0.87). (**C**) Model predictions are compared to transcription rate measurements for 10 promoters characterized by Hossain et al. using read-based measurements. (R^2 = 0.77, Spearman's ρ = 0.90). Dots and error bars represent the mean ± standard deviation for triplicate measurements (n = 3 biological replicates). Data are provided in **Supplementary Data 1**.

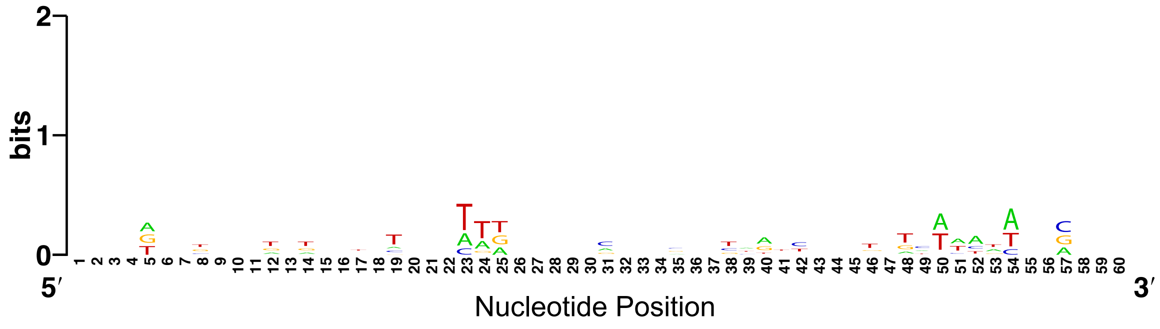

Supplementary Figure 6: Sequence Entropy for 60bp Forward Engineered Promoters. Sequence entropy analysis for 60 bp promoters showed that forward engineered promoters exhibit low levels of similarity. Promoters, on average, shared a ~27% sequence similarity and had an average pair-wise hamming distance of 44 bp. Data are provided in **Supplementary Data 1**.

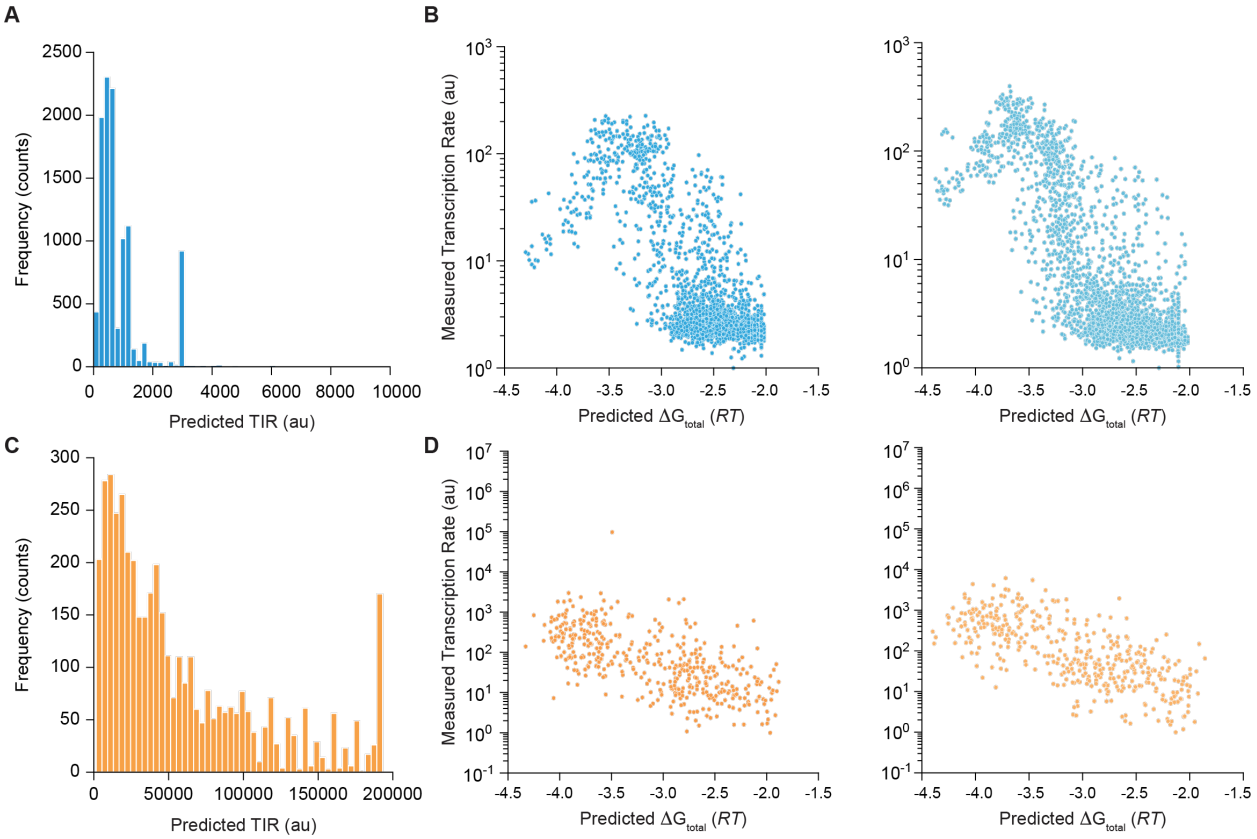

Supplementary Figure 7: mRNA Decay Alters RNA Level Measurements. (**A**) Predicted translation initiation rate (TIR) distribution for 10898 genome-integrated promoters, exhibiting a 1679-fold dynamic range. (**B**) Model predictions on 2716 low TIR promoters (left) and 2719 high TIR promoters (right). R^2 is equal to 0.51 (left) and 0.61 (right). (**C**) Predicted translation initiation rate (TIR) distribution for 4350 plasmid-endoded promoters, exhibiting a 2680-fold dynamic range. (**D**) Model predictions on 435 low TIR promoters (left) and 430 high TIR promoters (right). R^2 is equal to 0.43 (left) and 0.50 (right). Data are provided in **Supplementary Data 1**.

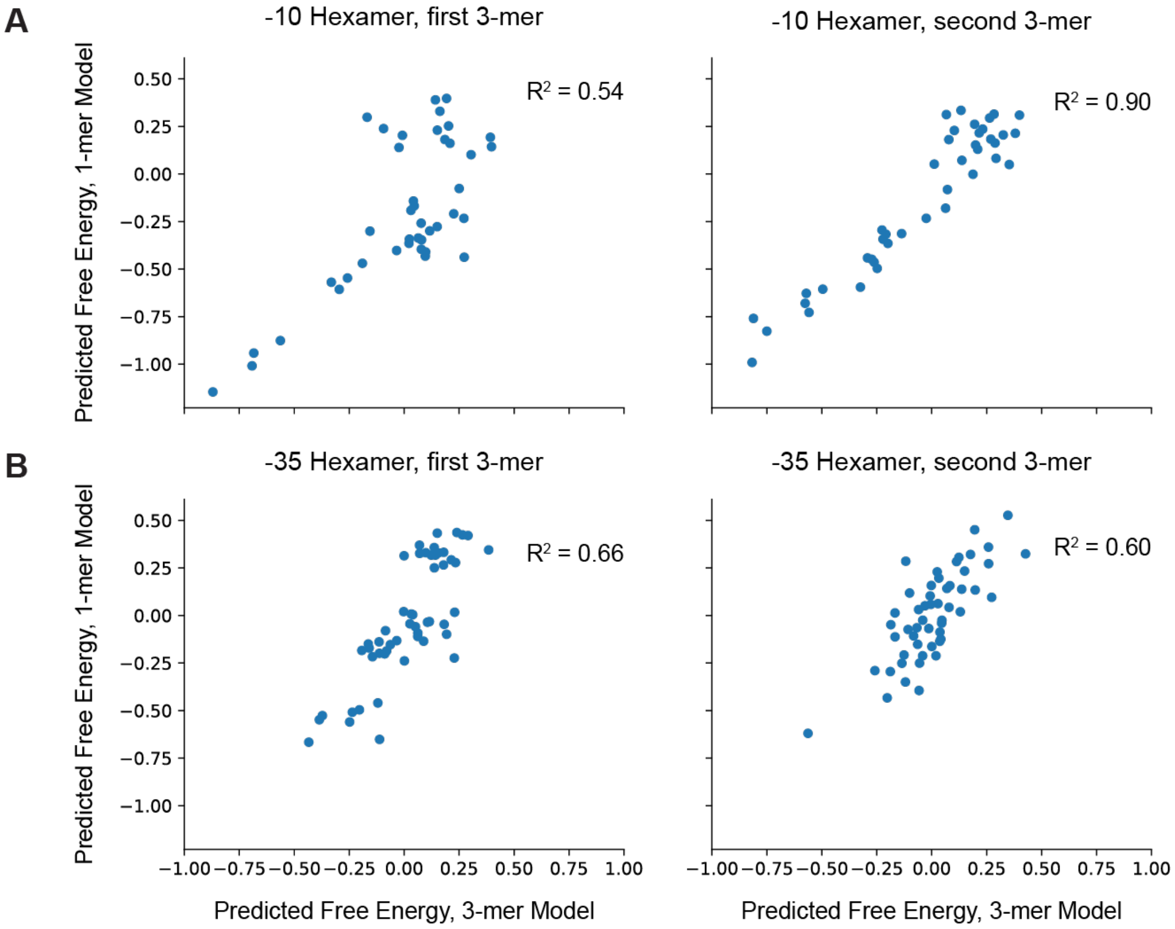

Supplementary Figure 8: Hexamer Model Comparison (1-mer vs. 3-mer). Correlation between predicted free energies of the single-nt model and tri-nt model for the (**A**) -10 hexamer and (**B**) -35 hexamer. For the single-nt model, 1-mer contributions are summed to calculate the free energy of each 3-mer, then plotted against model coefficients from the tri-nt model. Hexamers are broken up into 2 adjacent non-overlapping 3-mers. Squared Pearson correlation coefficients are reported. Data are provided in **Supplementary Data 4**.

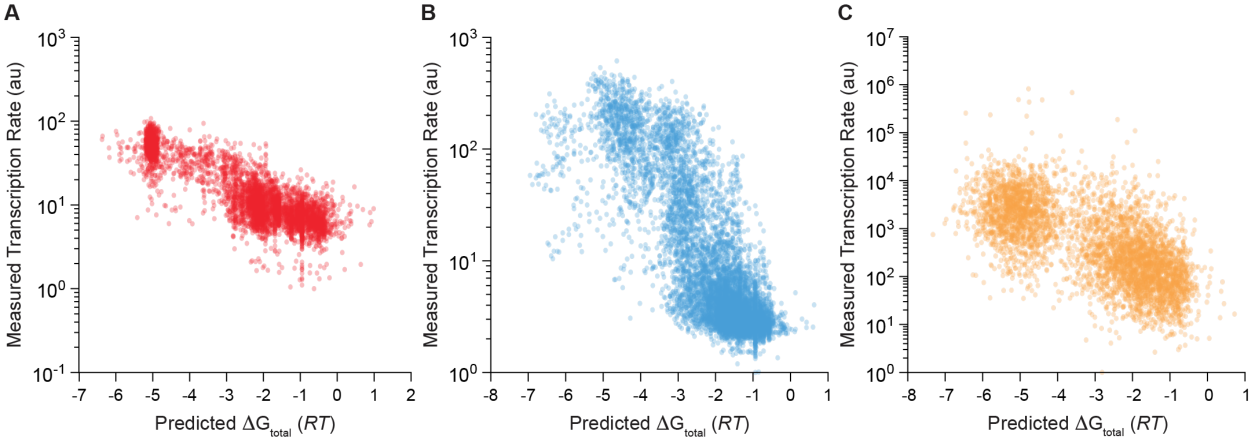

Supplementary Figure 9: A Quadratic Non-Linear Free Energy Model. (**A**) A non-linear free energy model incorporating all possible pair-wise interactions between promoter motifs was developed with 30 quadratic terms. Non-linear model predictions for 5391 designed promoters (this study) are compared to *in vitro* transcription rate measurements (R^2 = 0.76). (**B**) Non-linear model predictions for 10898 genome-integrated promoters are compared to *in vivo* transcription rate measurements (R^2 = 0.69). (**C**) Non-linear model predictions on 4350 non-repetitive plasmid-encoded promoters are compared to *in vivo* transcription rate measurements (R^2 = 0.46). Data are provided in **Supplementary Data 2**.

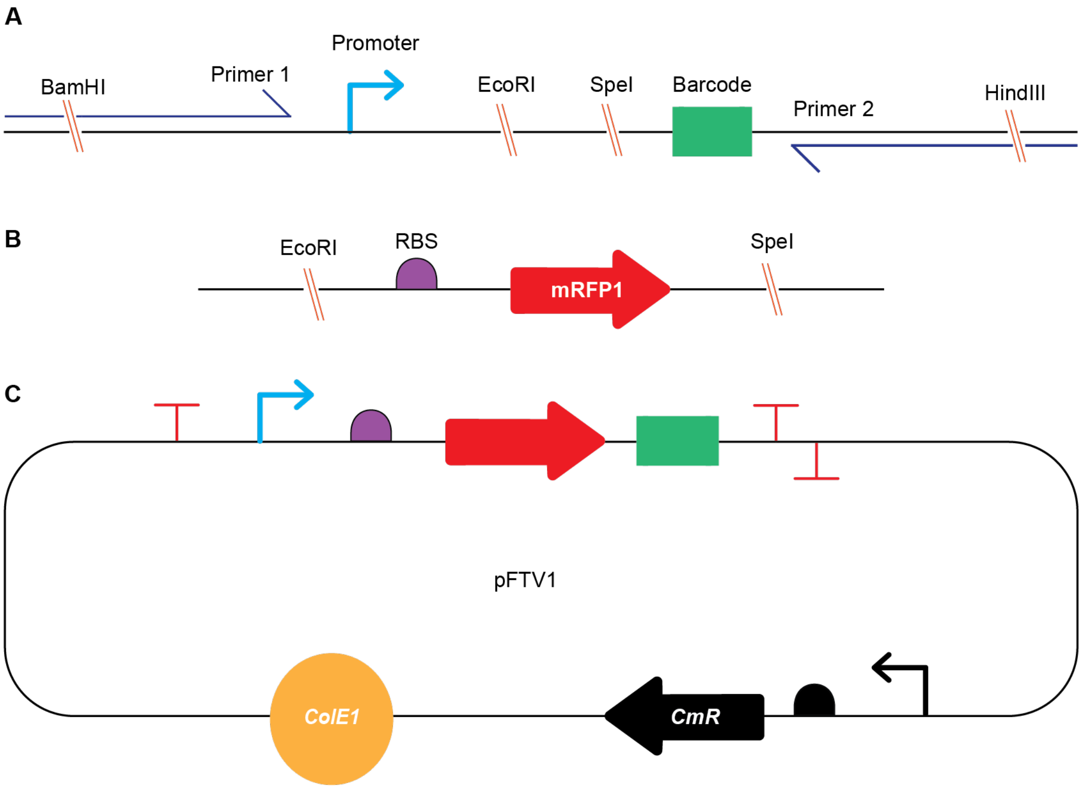

Supplementary Figure 10: Oligo Designs and Plasmid Map. (**A**) Oligopool library design with variable promoters. Each library variant contains two primer bindings sites with internal restriction enzyme cut sites (navy), a unique promoter, two additional restriction enzyme cut sites, and a unique barcode. (**B**) A Gene-block containing two restriction cut sites, a ribosome binding site (5k, RBS Calculator 2.1) and a reporter protein. (**C**) The final assembled plasmid pool used for promoter library characterization.

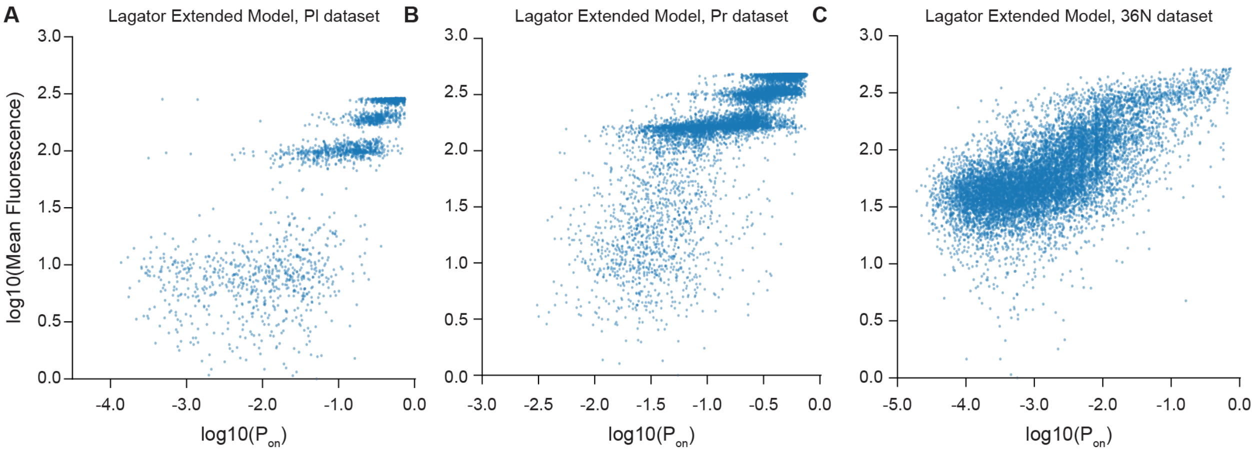

Supplementary Figure 11: Lagator Extended Model Predictions on Lagator Datasets. (**A**) Lagator extended model predictions for 2903 Pl mutant promoters are compared to reported *in vivo* Sort-Seq means (R^2 = 0.73). (**B**) Lagator extended model predictions for 12194 Pr mutant promoters are compared to reported *in vivo* Sort-Seq means (R^2 = 0.67). (**C**) Lagator extended model predictions for 11523 random 36N sequences are compared to reported *in vivo* Sort-Seq means (R^2 = 0.44). Data are provided in **Supplementary Data 3**.

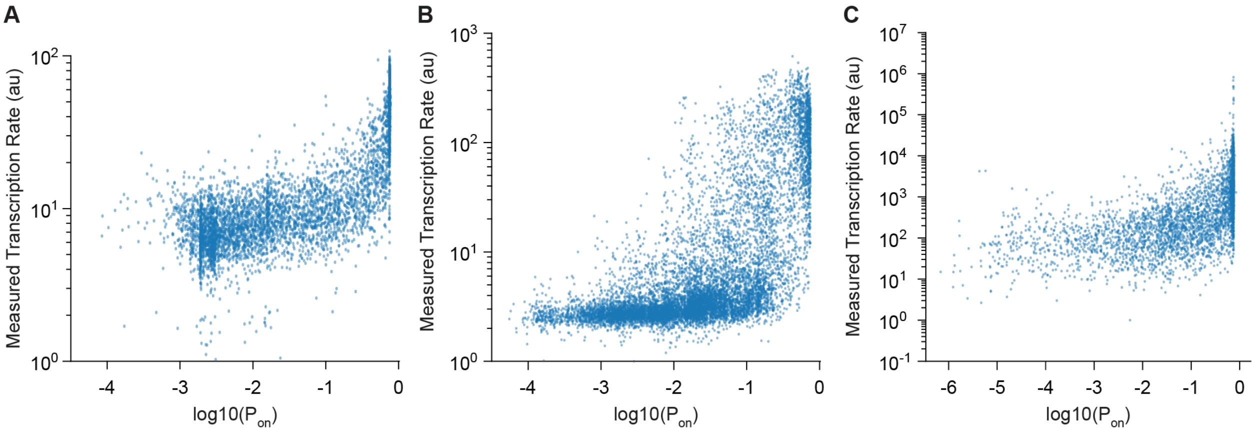

Supplementary Figure 12: Lagator Extended Model Validation. (**A**) Lagator extended model predictions for 5391 designed promoters (LaFleur et al., this study) are compared to *in vitro* transcription rate measurements (R^2 = 0.60). (**B**) Lagator extended model predictions for 10898 genome-integrated promoters (Urtecho et al.) are compared to *in vivo* transcription rate measurements (R^2 = 0.45). (**C**) Lagator extended model predictions on 4350 non-repetitive plasmid-encoded promoters (Hossain et al.) are compared to *in vivo* transcription rate measurements (R^2 = 0.39). Data are provided in **Supplementary Data 3**.

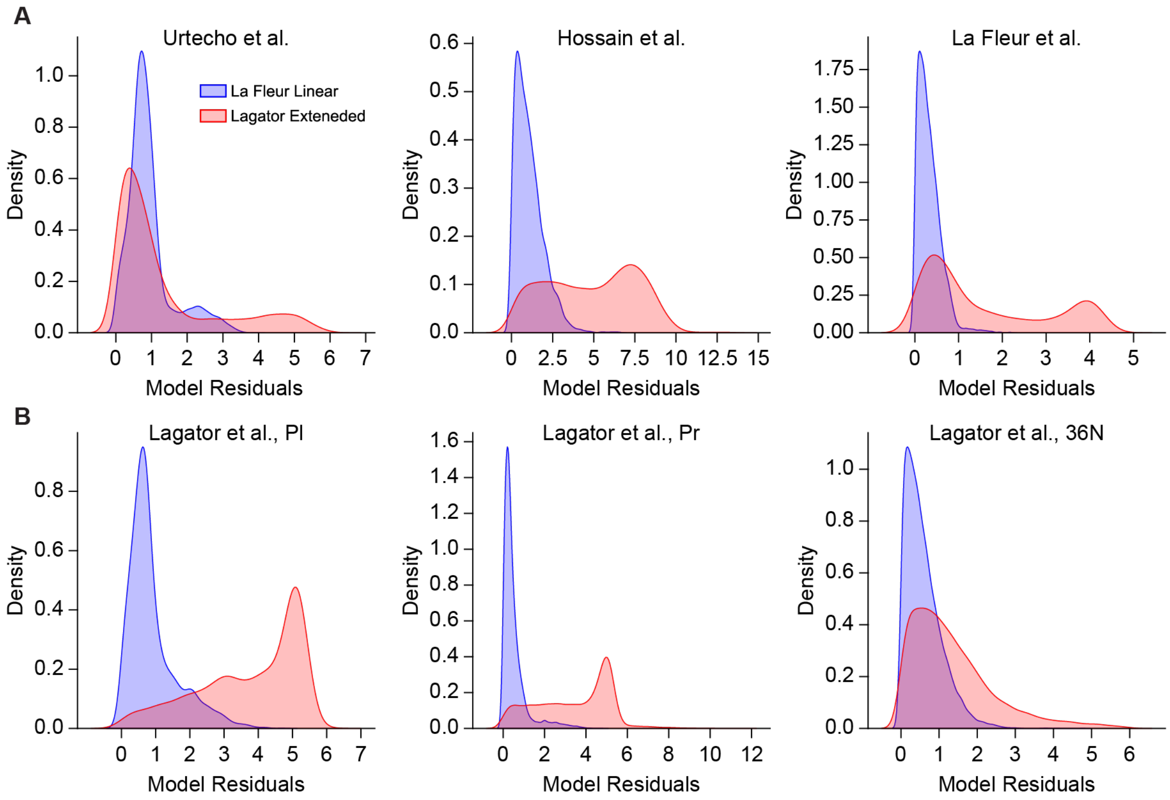

Supplementary Figure 13: Slope-only Model Benchmarking, Residual Distributions. (**A**) Model residual distributions for Urtecho et al. (left), Hossain et al. (middle) and LaFleur et al. (right). (**B**) Model residual distributions for the Pl dataset from Lagator et al. (left), Pr dataset from Lagator et al. (middle) and 36N dataset from Lagator et al. (right). Blue distributions are from the LaFleur et al. linear model, red distributions are from the Lagator et al. extended model. Model residuals are the absolute difference between the model prediction [ΔG_{total} for LaFleur and $\log(P_{on})$ for Lagator] and the natural log of the measured transcription rates, using a best-fit slope relationship for each model and dataset. Data are provided in **Supplementary Data 1 & 3**. Best fit parameters are provided in **Supplementary Data 5**.

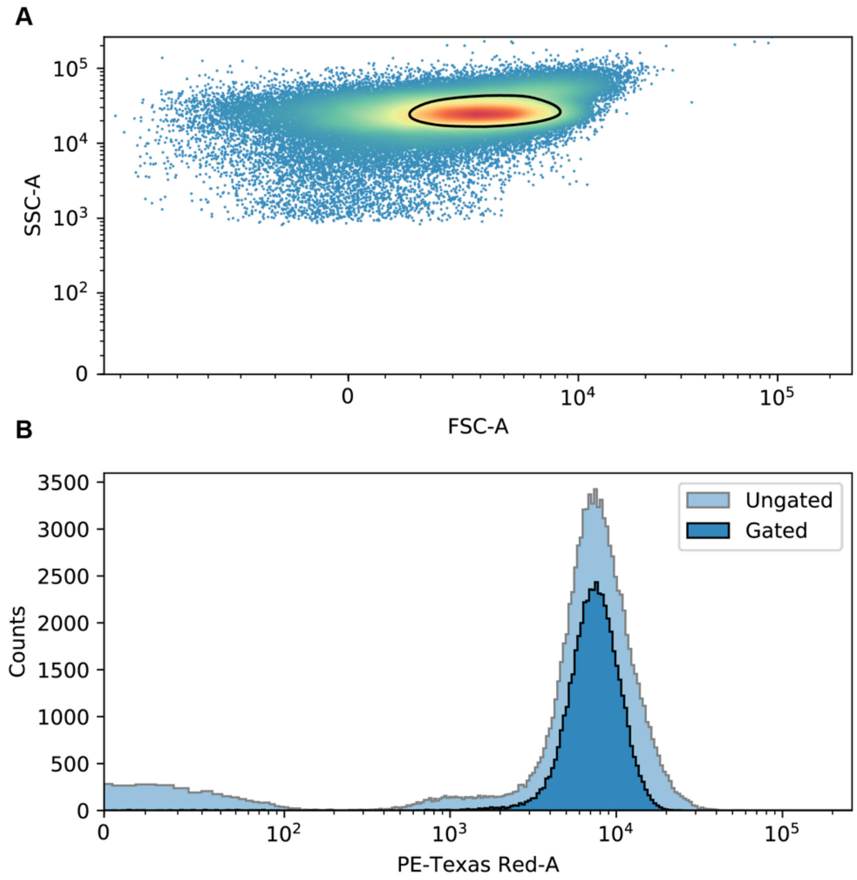

Supplementary Figure 14: Flow Cytometry Gating. (**A**) 2D density plot showing the gating strategy used in this study. Forward scattering and side scattering were used to identify events corresponding to *E. coli* cells, and the densest area (yellow and red) was used in calculating the mean fluorescence. (**B**) Texas Red intensity for the gated and ungated events.

Supplementary Table 1: Model Features.

A report of the total number of model features used for each promoter region, and the total number of features retained after feature reduction.

Feature Name, Feature Type	Number of Parameters	Number Retained[a]
-10 hexamer 3-mers	128	128
-35 hexamer 3-mers	128	128
Discriminator 3-mers	128	64
-10 extended 2-mers	32	16
Discriminator Length	7	0
Discriminator GC Content	1	0
Discriminator Purine Content	1	0
TSS 2-mers	16	0
Spacer Length	6	6
Spacer Stacking Free Energy	1	0
Spacer Torsional Energy	1	0
UP Groove Width (Distal and Proximal)	2	2
ITR Purine Content	1	0
ITR Pause-Element Location	15	0
UP A-tract Length (Distal and Proximal)	2	0
UP AT Content	1	0
DNA Rigidity	1	1
R-loop Strength	1	1

[a] *Retained parameters are the only ones present in the final model*

Supplementary Table 2: Model Selection.

A summary of the selection criteria used for converging on the final model version. Included are models with the original set of features (472) as well as models with the reduced set of features (346). Criteria for model selection was a high Pearson coefficient on the test set and 8 correct positive controls.

Model Type	Number of Features	R^2 Train	MAE Train	MSE Train	R^2 Test	MAE Test	MSE Test	Correct / Total Positive Controls[a]
Ridge	472	0.82	0.265	0.120	0.80	0.270	0.126	4/8
Lasso	472	0.82	0.264	0.120	0.80	0.270	0.126	4/8
Elastic Net	472	0.82	0.264	0.120	0.80	0.270	0.126	4/8
Ridge	346	0.80	0.274	0.133	0.80	0.281	0.131	8/8
Lasso	346	0.80	0.274	0.134	0.80	0.281	0.131	8/8
Elastic Net	346	0.80	0.274	0.133	0.80	0.281	0.131	8/8

[a] *Positive controls were defined to match known consensus promoter sequence motifs* [38]

Supplementary Table 3: Primers, RNA Adapter, and DNA Fragment Sequences.

A list of the nucleic acid sequences used during cloning.

Part Name	Part Sequence
primer 1	TGCTGGATCCCTACTCTGAG
primer 2	CTATAAGCTTGGTCCGACGG
primer 3	TCCCACAACGAAGACTACACC
primer 4	CTCTTTGATAACGTCTTCAGAGC
primer 5	AACAGGATCCACGCACTCTA
primer 6	CGGACGACCTTCACCTTCA
gene-block 1	CTGTGCGTTAGGTGATGCCGCGGCTGTACCGCCCATGTGCTCACGAATTCGAT<br>CAAATTTCGAGGTTCCATATGGCGAGCTCTGAAGACGTTATCAAAGAGTTCATG<br>CGTTTCAAAGTTCGTATGGAAGGTTCCGTTAACGGTCACGAGTTCGAAATCGAA<br>GGTGAAGGTGAAGGTCGTCCGTACGAAGGTACCCAGACCGCTAAACTGAAAGT<br>TACCAAAGGTGGTCCGCTGCCGTTCGCTTGGGACATCCTGTCCCCGCAGTTCC<br>AGTACGGTTCCAAAGCTTACGTTAAACACCCGGCTGACATCCCGGACTACCTG<br>AAACTGTCCTTCCCGGAAGGTTTCAAATGGGAACGTGTTATGAACTTCGAAGAC<br>GGTGGTGTTGTTACCGTTACCCAGGACTCCTCCCTGCAAGACGGTGAGTTCAT<br>CTACAAAGTTAAACTGCGTGGTACCAACTTCCCGTCCGACGGTCCGGTTATGCA<br>GAAAAAAACCATGGGTTGGGAAGCTTCCACCGAACGTATGTACCCGGAAGACG<br>GTGCTCTGAAAGGTGAAATCAAAATGCGTCTGAAACTGAAAGACGGTGGTCACT<br>ACGACGCTGAAGTTAAAACCACCTACATGGCTAAAAAACCGGTTCAGCTGCCG<br>GGTGCTTACAAAACCGACATCAAACTGGACATCACCTCCCACAACGAAGACTAC<br>ACCATCGTTGAACAGTACGAACGTGCTGAAGGTCGTCACTCCACCGGTGCTTA<br>ATAAACTAGTAAACGCAGTTACCCCATAGGCT
RNA adapter	AACAGGAUCCACGCACUCUANN

Supplementary Table 4: Model Benchmarking – Slope Only.

Model benchmarking with a best fit slope. Columns represent the LaFleur linear model, the LaFleur quadratic model, the Lagator standard model, the Lagator extended model, and the number of samples in each dataset. Rows correspond to performance metrics for each dataset.

	LaFleur (linear)	LaFleur (quadratic)	Lagator (standard)	Lagator (extended)	N samples
**R^2 Urtecho[a]**	0.60	0.69	0.47	0.45	10,898
**MAE Urtecho[a]**	0.93	0.56	1.32	1.33	
**MSE Urtecho[a]**	1.28	0.59	3.93	3.99	
**R^2 Hossain[a]**	0.45	0.46	0.42	0.39	4,350
**MAE Hossain[a]**	1.08	1.96	4.83	4.85	
**MSE Hossain[a]**	1.88	5.66	31.5	31.0	
**R^2 LaFleur**	0.79	0.76	0.58	0.60	5,391
**MAE LaFleur**	0.33	0.79	1.43	1.56	
**MSE LaFleur**	0.18	0.88	3.98	4.27	
**R^2 Lagator Pl**	0.47	0.49	0.67	0.73	2,903
**MAE Lagator Pl**	0.91	0.98	3.53	3.74	
**MSE Lagator Pl**	1.34	1.70	14.5	16.1	
**R^2 Lagator Pr**	0.35	0.37	0.55	0.67	12,194
**MAE Lagator Pr**	0.51	1.06	3.14	3.40	
**MSE Lagator Pr**	0.62	1.79	13.1	14.7	
**R^2 Lagator 36N**	0.19	0.15	0.33	0.44	11,523
**MAE Lagator 36N**	0.59	1.71	1.16	1.34	
**MSE Lagator 36N**	0.57	4.50	2.31	3.00	

[a] Rows with green shading indicate unseen datasets to both the LaFleur et al. and Lagator et al. models during training. See Discussion for details. Best fit parameters are provided in ***Supplementary Data 5***.

Supplementary Table 5: Model Benchmarking –Intercept Only.

Model benchmarking with a best fit intercept. Columns represent the LaFleur linear model, the LaFleur quadratic model, the Lagator standard model, the Lagator extended model, and the number of samples in each dataset. Rows correspond to performance metrics for each dataset.

	LaFleur (linear)	LaFleur (quadratic)	Lagator (standard)	Lagator (extended)	N samples
**R^2 Urtecho[a]**	0.60	0.69	0.47	0.45	10,898
**MAE Urtecho[a]**	0.82	0.56	1.95	1.24	
**MSE Urtecho[a]**	1.12	0.59	5.47	2.39	
**R^2 Hossain[a]**	0.45	0.46	0.42	0.39	4,350
**MAE Hossain[a]**	1.20	1.12	2.17	1.60	
**MSE Hossain[a]**	2.25	2.08	8.65	4.79	
**R^2 LaFleur**	0.79	0.76	0.58	0.60	5,391
**MAE LaFleur**	0.33	0.73	2.70	1.60	
**MSE LaFleur**	0.17	0.80	8.86	3.18	
**R^2 Lagator Pl**	0.47	0.49	0.67	0.73	2,903
**MAE Lagator Pl**	1.01	0.79	1.29	0.66	
**MSE Lagator Pl**	1.58	1.13	3.25	0.98	
**R^2 Lagator Pr**	0.35	0.37	0.55	0.67	12,194
**MAE Lagator Pr**	0.54	0.55	0.94	0.41	
**MSE Lagator Pr**	0.71	0.64	1.52	0.37	
**R^2 Lagator 36N**	0.19	0.15	0.33	0.44	11,523
**MAE Lagator 36N**	0.52	0.57	1.59	1.22	
**MSE Lagator 36N**	0.46	0.55	4.02	2.29	

[a] *Rows with green shading indicate unseen datasets to both the LaFleur et al. and Lagator et al. models during training. See Discussion for details. Best fit parameters are provided in* ***Supplementary Data 5****.*

Supplementary Table 6: Model Benchmarking – Slope and Intercept.

Model benchmarking with a best fit slope and intercept. Columns represent the LaFleur linear model, the LaFleur quadratic model, the Lagator standard model, the Lagator extended model, and the number of samples in each dataset. Rows correspond to performance metrics for each dataset.

	LaFleur (linear)	LaFleur (quadratic)	Lagator (standard)	Lagator (extended)	N samples
**R^2 Urtecho[a]**	0.60	0.69	0.47	0.45	10,898
**MAE Urtecho[a]**	0.68	0.56	0.78	0.81	
**MSE Urtecho[a]**	0.76	0.59	0.99	1.04	
**R^2 Hossain[a]**	0.45	0.46	0.42	0.39	4,350
**MAE Hossain[a]**	1.08	1.07	1.13	1.16	
**MSE Hossain[a]**	1.88	1.86	2.00	2.12	
**R^2 LaFleur**	0.79	0.76	0.58	0.60	5,391
**MAE LaFleur**	0.29	0.32	0.42	0.42	
**MSE LaFleur**	0.15	0.17	0.30	0.28	
**R^2 Lagator Pl**	0.47	0.49	0.67	0.73	2,903
**MAE Lagator Pl**	0.85	0.78	0.57	0.49	
**MSE Lagator Pl**	1.18	1.13	0.73	0.59	
**R^2 Lagator Pr**	0.35	0.37	0.55	0.67	12,194
**MAE Lagator Pr**	0.51	0.50	0.39	0.33	
**MSE Lagator Pr**	0.62	0.61	0.42	0.31	
**R^2 Lagator 36N**	0.19	0.15	0.33	0.44	11,523
**MAE Lagator 36N**	0.52	0.54	0.47	0.43	
**MSE Lagator 36N**	0.46	0.48	0.38	0.31	

[a] *Rows with green shading indicate unseen datasets to both the LaFleur et al. and Lagator et al. models during training. See Discussion for details. Best fit parameters are provided in* ***Supplementary Data 5****.*

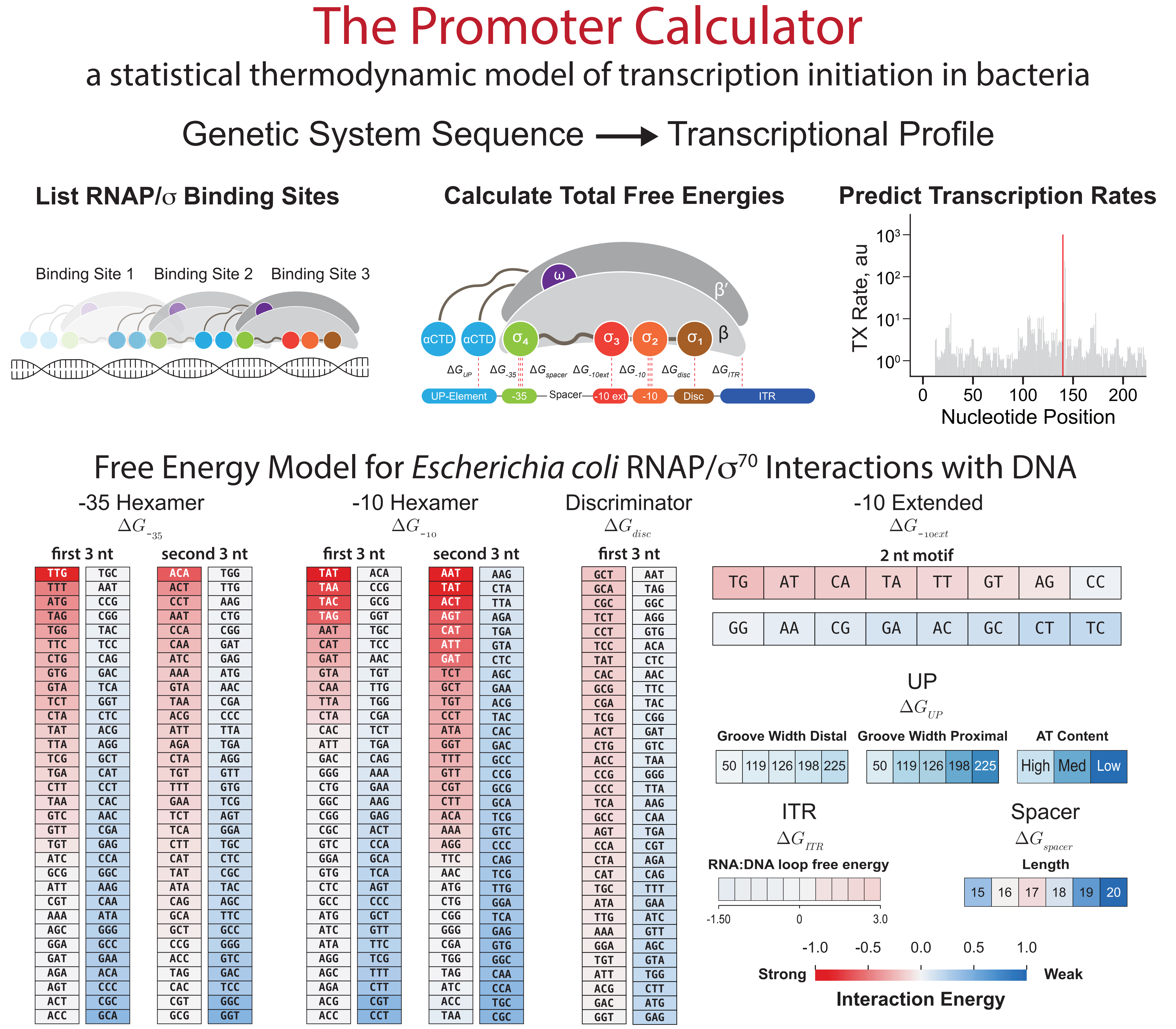

$$\Delta G_{total} = \Delta G_{UP} + \Delta G_{-35} + \Delta G_{spacer} + \Delta G_{-10ext} + \Delta G_{-10} + \Delta G_{disc} + \Delta G_{ITR}$$

$$TX = TX_{ref} \times e^{-\beta(\Delta G_{total} - \Delta G_{total,ref})}$$
