# Supplementary material for: Automated model-predictive design of synthetic promoters to control transcriptional profiles in bacteria
Source: Nat Commun. 2022 Sep 2;13:5159. doi: 10.1038/s41467-022-32829-5 (PMC9440211; doi:10.1038/s41467-022-32829-5)
Supplement: Supplementary file 3 — Reporting Summary [file 41467_2022_32829_MOESM3_ESM.pdf]

nature research

Corresponding author(s): Howard M. Salis

Last updated by author(s): 03/08/2022

Reporting Summary

Nature Research wishes to improve the reproducibility of the work that we publish. This form provides structure for consistency and transparency in reporting. For further information on Nature Research policies, see our Editorial Policies and the Editorial Policy Checklist.

Statistics

For all statistical analyses, confirm that the following items are present in the figure legend, table legend, main text, or Methods section.

n/a	Confirmed	
[ ]	[x]	The exact sample size (*n*) for each experimental group/condition, given as a discrete number and unit of measurement
[ ]	[x]	A statement on whether measurements were taken from distinct samples or whether the same sample was measured repeatedly
[ ]	[x]	The statistical test(s) used AND whether they are one- or two-sided<br>*Only common tests should be described solely by name; describe more complex techniques in the Methods section.*
[x]	[ ]	A description of all covariates tested
[ ]	[x]	A description of any assumptions or corrections, such as tests of normality and adjustment for multiple comparisons
[ ]	[x]	A full description of the statistical parameters including central tendency (e.g. means) or other basic estimates (e.g. regression coefficient) AND variation (e.g. standard deviation) or associated estimates of uncertainty (e.g. confidence intervals)
[ ]	[x]	For null hypothesis testing, the test statistic (e.g. *F*, *t*, *r*) with confidence intervals, effect sizes, degrees of freedom and *P* value noted<br>*Give P values as exact values whenever suitable.*
[x]	[ ]	For Bayesian analysis, information on the choice of priors and Markov chain Monte Carlo settings
[x]	[ ]	For hierarchical and complex designs, identification of the appropriate level for tests and full reporting of outcomes
[ ]	[x]	Estimates of effect sizes (e.g. Cohen's *d*, Pearson's *r*), indicating how they were calculated

Our web collection on statistics for biologists contains articles on many of the points above.

Software and code

Policy information about availability of computer code

Data collection	We used Python v3.7.7 and custom code (called the Oligopool Calculator v1.0) to carry out read processing, barcode mapping, barcode counting, and TSS identification. The Oligopool Calculator uses the following Python modules: numpy v1.19.0, biopython v1.77, leveldb v0.201, scipy v1.5.1, networkx v2.4, scikit-learn v0.23.1, seaborn v0.10.1, statsmodels v0.11.0, ssw-py v0.2.6
Data analysis	We used Python v3.7.7 and custom code that employed the following Python modules: sklearn v1.0.2, SciPy v1.7.3, numpy v1.21.2, pandas v1.3.5, matplotlib v3.5.0, pickle v4.0, FlowCal 1.2.2, itertools (Python v3.7.7). Source code is available at https://github.com/hsalis/SalisLabCode.

For manuscripts utilizing custom algorithms or software that are central to the research but not yet described in published literature, software must be made available to editors and reviewers. We strongly encourage code deposition in a community repository (e.g. GitHub). See the Nature Research guidelines for submitting code & software for further information.

Data

Policy information about availability of data

All manuscripts must include a data availability statement. This statement should provide the following information, where applicable:

- Accession codes, unique identifiers, or web links for publicly available datasets
- A list of figures that have associated raw data
- A description of any restrictions on data availability

Data and materials availability: All promoter sequences, model calculations, and experimental measurements are included in Supplementary Data. Next-generation sequencing read data files in fastQ format are available at NCBI with accession identifier PRJNA754118.

Field-specific reporting

Please select the one below that is the best fit for your research. If you are not sure, read the appropriate sections before making your selection.

- [x] Life sciences
- [ ] Behavioural & social sciences
- [ ] Ecological, evolutionary & environmental sciences

For a reference copy of the document with all sections, see nature.com/documents/nr-reporting-summary-flat.pdf

Life sciences study design

All studies must disclose on these points even when the disclosure is negative.

Sample size	Sample sizes for next-generation sequencing (RNA-Seq and DNA-Seq) were pre-determined (N = 3). A sample size of 3 was selected to gauge variability between in vitro replicates and reproducibility of the assay.
Data exclusions	All collected data is available in the Supplementary Data. Data filtering was carried out to train the model using promoters with single, predominant transcriptional start sites. The data filtering process is explicitly described ("Data Filtering").
Replication	Biological triplicates (N = 3) were carried out to quantify the reproducibility of transcription rate measurements (DNA-Seq and RNA-Seq read counts). For transcription rate measurements, biological replicates are independent in vitro transcription assays, followed by RNA harvest, amplicon library preparation, and next-generation sequencing.<br><br>Bilogical duplicates (N = 2), tripilicates (N =3), or quadruplicates (N =4) were performed for flow cytometry analysis. Separate isogenic colonies were harvested from agar plates and used to inoculate 96-well plates.<br><br>All replicates in these studies were reproducible.
Randomization	Randomization was not relevant for this study since each promoter was analyzed individually.
Blinding	Blinding was not relevant for this study since each promoter was analyzed individually.

Reporting for specific materials, systems and methods

We require information from authors about some types of materials, experimental systems and methods used in many studies. Here, indicate whether each material, system or method listed is relevant to your study. If you are not sure if a list item applies to your research, read the appropriate section before selecting a response.

Materials & experimental systems

n/a	Involved in the study	
x		Antibodies
x		Eukaryotic cell lines
x		Palaeontology and archaeology
x		Animals and other organisms
x		Human research participants
x		Clinical data
x		Dual use research of concern

Methods

n/a	Involved in the study	
x		ChIP-seq
	x	Flow cytometry
x		MRI-based neuroimaging

Flow Cytometry

Plots

Confirm that:

- [x] The axis labels state the marker and fluorochrome used (e.g. CD4-FITC).
- [x] The axis scales are clearly visible. Include numbers along axes only for bottom left plot of group (a 'group' is an analysis of identical markers).
- [x] All plots are contour plots with outliers or pseudocolor plots.
- [x] A numerical value for number of cells or percentage (with statistics) is provided.

Methodology

Sample preparation	10 ul bacterial cell aliquots were extracted and added to 190 ul PBS with 2mg/mL kanamycin to stop protein production. Flow cytometry was then carried out on the fixed cells to record their fluorescence distribution (BD LSR Fortessa). The mRFP1 fluorescence level was the arithmetic mean of the measured fluorescence distribution subtracted by autofluorescence, which

	was the arithmetic mean of the fluorescence distribution of wild-type E. coli DH10B cells.
Instrument	BD LSR Fortessa
Software	FlowCal software was used to process flow cytometry data.
Cell population abundance	Over 30000 post-sort events were collected. All post-sort cell populations were single clusters. All post-sort fluorescence distributions were unimodal (single peaked).
Gating strategy	Gates included: forward scatter measurements must be within [100,10000]; side scatter measurements must be within [6000, 55000]; fluorescence measurements must be 0 or greater.

☒ Tick this box to confirm that a figure exemplifying the gating strategy is provided in the Supplementary Information.
