# Supplementary material for: Automated model-predictive design of synthetic promoters to control transcriptional profiles in bacteria
Source: Nat Commun. 2022 Sep 2;13:5159. doi: 10.1038/s41467-022-32829-5 (PMC9440211; doi:10.1038/s41467-022-32829-5)
Supplement: Supplementary file 4 — Description of Additional Supplementary Files [file 41467_2022_32829_MOESM4_ESM.pdf]

Title: Supplementary Data 1
Description: Sequences, measurements, and LaFleur linear model predictions across all datasets

Title: Supplementary Data 2
Description: Sequences and LaFleur quadratic model predictions across all datasets.

Title: Supplementary Data 3
Description: Lagator et al. extended model predictions and sequences.

Title: Supplementary Data 4
Description: F-tests, model benchmarking, and mono-nt vs. tri-nt model comparison.

Title: Supplementary Data 5
Description: Fitted model slopes and intercepts used for benchmarking.
